# Prevalence of medication adherence and glycemic control among patients with type 2 diabetes and influencing factors: A cross-sectional study

**DOI:** 10.1016/j.gloepi.2023.100113

**Published:** 2023-06-10

**Authors:** Budi Suprapti, Zamrotul Izzah, Ade Giriayu Anjani, Mareta Rindang Andarsari, Wenny Putri Nilamsari, Cahyo Wibisono Nugroho

**Affiliations:** a Department of Pharmacy Practice, Faculty of Pharmacy Universitas Airlangga, Nanizar Zaman Joenoes Building, Campus C Unair Mulyorejo, Surabaya 60115, Indonesia; b Department of Pharmacy, Universitas Airlangga Hospital, Campus C Unair Mulyorejo, Surabaya 60115, Indonesia; c Department of Pharmaceutical Analysis, Groningen Research Institute of Pharmacy, University of Groningen, Antonius Deusinglaan 1, 9713 AV Groningen, Netherlands; d Master of Clinical Pharmacy Program, Faculty of Pharmacy Universitas Airlangga, Nanizar Zaman Joenoes Building, Campus C Unair Mulyorejo, Surabaya 60115, Indonesia; e Department of Internal Medicine, Faculty of Medicine Universitas Airlangga, Mayjen Prof. Dr. Moestopo 47, Surabaya 60131, Indonesia; f Department of Internal Medicine, Universitas Airlangga Hospital, Campus C Unair Mulyorejo, Surabaya 60115, Indonesia

**Keywords:** Brief Medication Questionnaire, Glycosylated hemoglobin, Indonesia, Medication adherence, Type 2 diabetes

## Abstract

**Background:**

This study aimed to assess medication adherence, glycemic control, and their influencing factors among outpatients at an Indonesian clinic with type 2 diabetes.

**Methods:**

A cross-sectional study was conducted among patients with type 2 diabetes at a hospital-based clinic in Surabaya, Indonesia, from September to December 2018. A purposive sampling was used; patients aged 18 years and older, had diabetes and any comorbidity, received hypoglycemic agents, and provided written informed consent were included. The previously validated Brief Medication Questionnaire was used to measure medication adherence, while glycosylated hemoglobin (A1C) levels were used to evaluate glycemic control. Binary logistic regression was used to identify factors associated with medication adherence and glycemic control.

**Results:**

Of 321 patients enrolled in the study, 268 (83.5%) patients were medication nonadherent. Patients who did not engage regularly in physical activity (aOR: 0.49, 95% CI: 0.26–0.93) was more likely to be medication adherent. Poor glycemic control (A1C: >7%) was observed in 106 (33.0%) of the patients. Patients who used a combination of oral hypoglycemic agents and insulin (aOR: 2.74, 95% CI: 1.09–6.86), did not take biguanide (aOR: 2.73, 95% CI: 1.16–6.43), reported hyperglycemia (aOR: 4.24, 95% CI: 1.53–11.81), and had comorbid diseases (aOR: 4.33, 95% CI: 1.08–17.34) increased the risk of having poor glycemic control. Patients who were more likely to achieve good glycemic control were male (aOR: 0.39, 95% CI: 0.20–0.74) and aged older (aOR: 0.95, 95% CI: 0.92–0.99).

**Conclusions:**

The proportion of patients who were medication nonadherent was much higher than those with poor glycemic control. Whereas regular exercise was a predictor of nonadherence, age, sex, diabetes medication, not taking biguanide, acute complications, and comorbidity were predictors of poor glycemic control. Therefore, strategies are needed to improve medication adherence and glycemic control.

## Introduction

The global prevalence of diabetes is rising at an alarming rate. In 2021, the estimated number of people aged 20–79 years with diabetes worldwide was 536.6 million, with this figure projected to rise to 783.2 million by 2045 [1,2]. Therefore, diabetes is a serious concern requiring the attention of the public, healthcare professionals, and policy and decision makers. Those with diabetes are at risk of developing complications caused by uncontrolled blood glucose levels, which lead to a lower quality of life and premature death. Diabetes cannot be controlled solely by taking medication. Other self-care approaches, including dietary restrictions, regular physical activity, routine foot care, and self-monitoring of blood glucose levels, are recommended for lowering the incidence and progression of complications [3].

Indonesia ranks fifth globally for the highest number of individuals with diabetes aged 20–79 years, estimated to be 19.5 million in 2021 and projected to rise to 28.6 million in 2045 [2]. Most diabetes-related services and treatments in Indonesia are covered under Jaminan Kesehatan Nasional (the national health insurance scheme). The majority of patients receive their initial treatment at *puskesmas* (primary health centers), which serve as the first point of contact for the provision of care within the public health system [4]. Patients with diabetes-related complications who require complex interventions and/or have problems taking medication for various reasons are commonly referred to hospitals. Adherence to the prescribed diabetes treatment is necessary to slow down the progression of the disease and decrease the risk of morbidity and mortality [5]. However, medication adherence in general is a complex behavior, and influencing factors vary according to the patients' knowledge, beliefs, social and economic status, disease characteristics, type of therapy, and the healthcare system [3,5,6].

Achieving good glycemic control is often challenging in practice. Studies conducted in Asian countries have found that >60% of treated diabetics had poor glycemic control, as verified by fasting plasma glucose or glycosylated hemoglobin (A1C) measurements [7,8]. Barriers associated with a complex regimen, such as fear of adverse drug reactions, hypoglycemia, and adherence problems, may hinder effective management of diabetes. In the specific context of Indonesia, despite the high prevalence of diabetes, this topic remains underexplored. Given that medication nonadherence and uncontrolled blood glucose levels can cause diabetes-related complications and death, there is a critical need for assessments of diabetes patients' medication adherence, glycemic control, and their influencing factors. Inputs from such assessments can be used by healthcare providers to formulate strategies for improving clinical and patient health outcomes relating to type 2 diabetes.

## Methods

### Study design

Between September and December 2018, a cross-sectional study was conducted among adults with type 2 diabetes being treated at the internal medicine clinic of a secondary referral hospital in Surabaya, Indonesia. The study protocol was reviewed and approved by the ethics research committee at Universitas Airlangga Hospital in Surabaya, Indonesia (approval number: 173/KEH/2018 dated July 31, 2018). The study also adheres to the principles of the Declaration of Helsinki. Patients provided written informed consent prior to participating in the study.

The sample size was calculated based on a single population proportion formula [9] by considering the proportion 60% with a 95% confidence interval and a 5% margin of error, thus the sample size was 369. Since the sample was taken from the total population of 1200 patients with type 2 diabetes visiting the clinic, the minimum sample size was adjusted to 292 [10]. Finally, with the addition of 10% withdrawal, at least 321 patients were recruited.

Inclusion criteria for the study were patients with a type 2 diabetes diagnosis who were aged 18 years and older and under treatment with diabetes medication (mono or combination therapy). Those with any comorbidity were also included in the study. A purposive sampling method was used; the investigators approached patients visiting the clinic individually, described the study, and invited them to participate voluntarily in this study. They were requested to provide written informed consent if they agreed to enroll in the study. There were no exclusion criteria; however, their data were removed from the analysis if they withdrew from the study. Furthermore, the study participants did not receive any financial compensation.

### Data collection

The patients' details were fully anonymized. Demographic and clinical characteristics, namely age, sex, education level, the lapse of time since receiving a type 2 diabetes diagnosis, comorbid disease(s), complication(s), body mass index, and blood glucose and A1C measurements were retrieved from the clinic's medical records. Details regarding the drug therapies administered to patients were obtained from the clinic's pharmacy records. These records were obtained with the patients' permission. In addition, patients were interviewed using an evidence-based checklist for the detection of drug-related problems in type 2 diabetes [11]. The interviews yielded further information on their diets, levels of physical activity (at least 150 min of physical activity per week was considered “regular”), smoking habits, and medication use.

Adherence to medication was assessed using the validated Brief Medication Questionnaire (BMQ) [[12], [13], [14], [15]]. A forward–backward translation protocol was applied prior to data collection to develop a Bahasa Indonesia version of the BMQ (Supplementary Text) derived from the original English version. Permission to use the questionnaire was granted by its original authors. The forward translation into Bahasa Indonesia was handled by two Indonesian translators with advanced English skills. The translations were then compared and discussed to reach an agreement. The Bahasa Indonesia version was then translated back into English by a native English speaker fluent in Bahasa Indonesia. Following comparison and discussion of the back translation and the original English version, the meaning of all questions in both versions were found to be the same. A pre-testing of the Bahasa Indonesia version was administered to 10 patients with type 2 diabetes from the same clinic in August 2018. The value of Cronbach's alpha coefficient for the Bahasa Indonesia version of BMQ was 0.742 (>0.70) and the correlation coefficient (*r*-value >0.80), which met the criteria of reliability and construct validity (data not shown).

The BMQ comprises three screens: regimen, belief, and recall [12]. The regimen screen presents seven items on the presence or absence of any self-reported nonadherence to the current drug regimen taken in the past week. Indicators of nonadherence included failing to mention the medication, reporting any discontinuation due to a late refill or other reason, reporting any missed doses, or reporting any extra doses. The belief screen comprised two items on any negative belief or belief barriers regarding efficacy, side effects, or other concerns regarding a specific drug that was perceived as bothersome. The recall screen presented two items on any recall barrier or difficulties in remembering to take medication. Each response was assigned a score of 0 (“No”), indicating an absence of any self-reported nonadherence, or 1 (“Yes”), indicating self-reported nonadherence or the presence of any barriers. The level of medication adherence was evaluated according to the positive responses for the three screens: adherence (no positive response for any of the three screens), probable adherence (a positive response for one screen), probable low adherence (positive responses for two screens), and low adherence (positive responses for all three screens). To facilitate interpretation, adherence and probable adherence were combined into one category of adherence, while probable low and low adherence were combined into one category of nonadherence.

Glycemic control was evaluated using the patients' recent A1C measurements. Glycemic control was classified as “good” (A1C: ≤7%) or “poor” (A1C: >7%) for all patients as per the therapeutic target levels stated in the clinical guidelines applied at the study site.

### Data analysis

All data were tabulated and coded using Microsoft Excel (2016). The dependent variables were adherence level (adherent or nonadherent) and glycemic control (good or poor). The independent variables included age (in years), sex (female or male), body mass index (<25.0 or ≥ 25.0 kg/m^2^), education level (primary, secondary, or tertiary), currently smoking (yes or no), dietary adjustments (yes or no), regular physical activity (yes or no), duration of type 2 diabetes (in years), number of prescribed medications (<5 or ≥ 5; the latter refers to polypharmacy), diabetes medication (monotherapy, combination of oral hypoglycemic agents, or combination of oral hypoglycemic agents and insulin), use of hypoglycemic agent (insulin, biguanide, sulphonylurea, alpha glucosidase inhibitor, and thiazolidinedione), use of other medications (e.g. antihypertensive agent, antihyperlipidemic drug, and platelet-aggregation inhibitor), acute complication (hypoglycemia, hyperglycemia, or both), and comorbidity. The adherence level was also considered as a predictor when analyzing the glycemic outcome.

The statistical analysis was performed using the SPSS statistical program, v. 28.0 for Windows (IBM Corp, Armonk, New York). Descriptive statistics were used to describe patients' characteristics, levels of adherence to medication, and the prevalence of glycemic control. The Kolmogorov-Smirnov test and a Q-Q plot were performed to evaluate whether the continuous variables had a normal distribution. Data were presented in numbers, percentages, and means ± standard deviations (SDs) or as medians ± interquartile ranges (IQRs), if applicable.

Bivariate and multivariable logistic regression analyses were performed to examine factors associated with medication nonadherence and poor glycemic control as well as the association between nonadherence to medication and poor glycemic control. The linearity assumption underlying logistic regression model for continuous predictors was previously tested using the Box-Tidwell test and a scatter plot, showing that the predictors were linearly related to the logit of the outcome variable. Finally, the results of the logistic regression analyses were presented as odds ratios (ORs) for the bivariate analysis and adjusted odds ratios (aORs) for the multivariable analysis, with a 95% confidence interval (CI).

The final multivariable models were adjusted for previously known variables associated with adherence and glycemic control (i.e., age, duration of type 2 diabetes, diabetes medication, acute complication, and comorbidity) and completed with variables showing a trend toward association in bivariate analysis. These variables were selected using a backward elimination approach with the alpha level of 0.05, and the final models retained all additional variables with *p* < 0.25. The model's performance with regard to its calibration and discrimination was examined. The Hosmer and Lemeshow “goodness-of-fit” test was used to assess the model's calibration, while the C statistic, area under the receiver operating characteristic curve, was used to evaluate its discrimination [16]. This study's reporting procedure conforms to the STROBE guidelines [17].

## Results

A total of 321 patients participated in the study. None of them withdrew their participation after enrollment. Table 1 presents the patients' demographic and clinical characteristics. The continuous variables (i.e., age and duration of type 2 diabetes) were not normally distributed as shown by the Kolmogorov-Smirnov test (*p* < 0.05) and the Q-Q plots (the data points deviated from the diagonal line), thus they were presented in medians and IQRs. Of the total patients, a median (IQR) age was 61.0 (54.0–67.0) years and 171 (53.3%) were female patients. A total of 248 (77.3%) patients had minimally a secondary level of education. The median (IQR) duration of type 2 diabetes was 8.0 (3.0–15.0) years. Of the patients who engaged in self-care behavior, 192 (59.8%) engaged in regular exercise, 270 (84.1%) controlled their diet, and 296 (92.2%) were nonsmokers. A total of 217 patients (67.6%) used a combination of hypoglycemic agents for their treatment. Of these hypoglycemic agents, insulin was most commonly administered to patients (*n* = 191; 59.5%) followed by oral hypoglycemic agents, specifically biguanide (*n* = 179; 55.8%) and sulphonylurea (*n* = 170; 53.0%). Approximately 30% of patients reported acute complications of hypoglycemia or hyperglycemia. Only 27 patients (8.4%) were diagnosed with diabetes without any comorbidities, with the remaining patients evidencing diabetes with comorbidities such as hypertension (*n* = 188; 58.6%), gastrointestinal disorders (*n* = 70; 21.8%), and coronary heart disease (*n* = 69; 21.5%).

**Table 1:** Patients' characteristics (*N* = 321).

Variable	*n* (%)
Age (years)	
Range (min, max)	28.0–87.0
Median (IQR)	61.0 (54.0–67.0)

Sex	
Female	171 (53.3)
Male	150 (46.7)

Body mass index (kg/m^2^)	
<25.0	122 (38.1)
≥25.0	134 (41.7)
No records	65 (20.2)

Education level	
Tertiary	68 (21.2)
Secondary	180 (56.1)
Primary	63 (19.6)
No records	10 (3.1)

Dietary adjustments	
Yes	270 (84.1)
No	51 (15.9)

Regular physical activity	
Yes	192 (59.8)
No	129 (40.2)

Currently smoking	
Yes	25 (7.8)
No	296 (92.2)

Duration of type 2 diabetes (years)	
Range (min, max)	0.1–36.0
Median (IQR)	8.0 (3.0–15.0)

Number of prescribed medications	
<5	203 (63.2)
≥5 (polypharmacy)	118 (36.8)

Diabetes medication	
Monotherapy	104 (32.4)
Combination of oral hypoglycemic agents	103 (32.1)
Combination of oral hypoglycemic agents and insulin	114 (35.5)

Type of hypoglycemic agent	
Insulin	191 (59.5)
Biguanide	179 (55.8)
Sulphonylurea	170 (53.0)
Alpha-glucosidase inhibitor	82 (25.5)
Thiazolidinedione	7 (2.2)

Other medications	
Antihypertensive agents	185 (57.6)
Antihyperlipidemic drugs	164 (51.1)
Platelet-aggregation inhibitors	84 (26.2)
Antihyperuricemic drugs	42 (13.1)
Neuropathic pain medications	34 (10.6)
Analgesics	26 (8.1)
Antianginal drugs	23 (7.2)

Acute complication	
Hypoglycemia	55 (17.1)
Hyperglycemia	36 (11.2)
Both hypo- and hyperglycemia	3 (0.9)
No	227 (70.7)

Presence of comorbidity	
Yes	294 (91.6)
No	27 (8.4)

Comorbid disease^a^	
Hypertension	188 (58.6)
Gastrointestinal disorder	70 (21.8)
Coronary heart disease	69 (21.5)
Chronic kidney disease	41 (12.8)
Neurological disorder	19 (5.9)
Respiratory disease	14 (4.4)
Dyslipidemia	8 (2.5)
Hepatic disorder	5 (1.6)
Hyperuricemia	4 (1.2)
Eye disorders	4 (1.2)
Benign prostate hyperplasia	3 (0.9)

Note: IQR = interquartile range.

a Subject could have one or more comorbidities.

The proportion of nonadherent patients observed in this study was high (*n* = 268; 83.5%) (Table 2). The majority of the patients reported noncompliance (positive responses) for the regimen screen and the recall screen (barriers to drug use), including side effects, difficulties opening the drug container, and difficulty remembering all the doses to be taken and taking many pills at the same time. The results of the bivariate analysis (Supplementary Table S1) showed that the risk of nonadherence to medication was less likely to be associated with the absence of regular physical activity (OR: 0.54, 95% CI: 0.30–0.98). In the final multivariable analysis (Table 3), regular physical activity was associated with nonadherence to medication. Patients who did not engage in regular exercise were 0.5 times less likely to be nonadherent (aOR: 0.49, 95% CI: 0.26–0.93) than those who engaged regularly in physical activity.

**Table 2:** Adherence to medications among outpatients with type 2 diabetes (*N* = 321).

Variable	*n* (%)
Drug use compliance	
Regimen screen	
No positive response	63 (19.6)
Positive response (noncompliant)	258 (80.4)

Barriers to drug use	
Belief screen	
No positive response	243 (75.7)
Positive response (barriers present)	78 (24.3)
Recall screen	
No positive response	1 (0.3)
Positive response (barriers present)	320 (99.7)

Adherence	
Adherent (no positive response for the three screens)	0 (0)
Probable adherence (positive response for one screen)	53 (16.5)
Probable low adherence (positive responses for two screens)	201 (62.6)
Low adherence (positive responses for all three screens)	67 (20.9)

**Table 3:** Factors associated with medication nonadherence among outpatients with type 2 diabetes.

Variable	Adherence level, *n* (%)		OR (95% CI)	Adjusted OR (95% CI)
	Adherent	Nonadherent		
Age (years)	53 (16.5)	268 (83.5)	0.99 (0.96–1.02)	0.99 (0.96–1.03)

Education level				
Tertiary	15 (4.8)	53 (17.0)	1.00	
Secondary	28 (9.0)	152 (48.9)	1.54 (0.76–3.10)	2.31 (0.86–6.23)
Primary	8 (2.6)	55 (17.7)	1.95 (0.76–4.97)	1.93 (0.90–4.13)

Regular physical activity				
Yes	25 (7.8)	167 (52.0)	1.00	
No	28 (8.7)	101 (31.5)	0.54 (0.30–0.98)	0.49 (0.26–0.93)

Duration of type 2 diabetes (years)	53 (16.5)	268 (83.5)	0.99 (0.96–1.03)	1.00 (0.96–1.04)

Diabetes medication				
Monotherapy	16 (5.0)	88 (27.4)	1.00	
Combination of oral hypoglycemic agents	12 (3.7)	91 (28.3)	1.38 (0.62–3.08)	1.37 (0.56–3.36)
Combination of oral hypoglycemic agents and insulin	25 (7.8)	89 (27.7)	0.65 (0.32–1.30)	0.60 (0.28–1.26)

Use of thiazolidinedione				
Yes	3 (0.9)	4 (1.2)	0.25 (0.06–1.16)	0.20 (0.03–1.23)
No	50 (15.6)	264 (82.2)	1.00	

Use of an antihypertensive agent				
Yes	31 (9.7)	154 (48.0)	0.96 (0.53–1.74)	1.29 (0.67–2.47)
No	22 (6.9)	114 (35.5)	1.00	

Use of an antihyperlipidemic drug				
Yes	31 (9.7)	133 (41.4)	0.70 (0.39–1.27)	0.81 (0.43–1.55)
No	22 (6.9)	135 (42.1)	1.00	

Acute complication				
Hypoglycemia	12 (3.7)	43 (13.4)	0.68 (0.33–1.41)	0.78 (0.33–1.83)
Hyperglycemia	4 (1.2)	32 (10.0)	1.51 (0.50–4.52)	1.83 (0.55–6.11)
Both hypo- and hyperglycemia	1 (0.3)	2 (0.6)	0.38 (0.03–4.27)	0.47 (0.04–5.63)
No	36 (11.2)	191 (59.5)	1.00	

Presence of one or more comorbidities				
Yes	51 (15.9)	243 (75.7)	0.38 (0.09–1.66)	0.16 (0.02–1.29)
No	2 (0.6)	25 (7.8)	1.00	

Notes: Adherence data from 321 patients were analyzed. The results of the Hosmer and Lemeshow “goodness-of-fit” test of the final model showed χ^2^ = 2.75 with degrees of freedom = 8. The model's discrimination, measured as the area under the receiver operating characteristic curve, was 0.71 (95% CI: 0.63–0.79).

CI = confidence interval; OR = odds ratio.

**Fig. 1:**
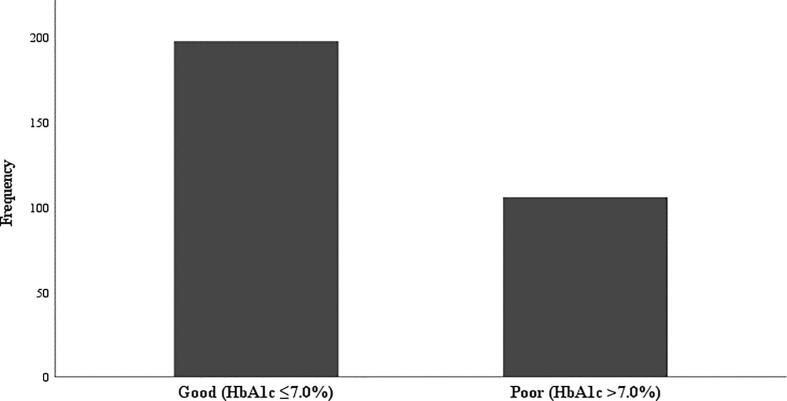
Glycemic targets of outpatients at an Indonesian clinic with type 2 diabetes and their profiles.

As illustrated in Fig. 1, the prevalence of patients with poor glycemic control was lower (*n* = 106; 33.0%) than that of patients with good glycemic control (*n* = 198; 61.7%). A1C measurements could not be retrieved from 17 (5.3%) patients' medical records. Supplementary Table S2 presents the results of the bivariate analysis. They reveal that the following factors were associated with good glycemic control: the age of a patient (OR: 0.95, 95% CI: 0.93–0.98), being male (OR: 0.56, 95% CI: 0.34–0.91), and nonuse of insulin (OR: 0.52, 95% CI: 0.32–0.86). Poor glycemic control was more likely to occur among patients who did not take biguanide (OR: 1.62, 95% CI: 1.01–2.60) and those who experienced hyperglycemia (OR: 4.97, 95% CI: 2.27–10.85). Being nonadherent to medication was not associated with poor glycemic control (OR: 0.97, 95% CI: 0.51–1.85). The results of the final multivariable analysis (Table 4) showed that diabetes medication, biguanide use, presence of hyperglycemia, and comorbidity were associated with poor glycemic control. The risk of poor glycemic outcome was 2.7 times higher among patients who were receiving a combination of oral hypoglycemic agents and insulin compared with those receiving monotherapy (aOR: 2.74, 95% CI: 1.09–6.86). Patients who were not taking biguanide for their diabetes treatment were 2.7 times more likely to have poor glycemic control (aOR: 2.73, 95% CI: 1.16–6.43). Patients who reported hyperglycemia during the treatment were 4.2 times (aOR: 4.24, 95% CI: 1.53–11.81) more likely to have poorly controlled A1C levels compared with those who did not report any acute complications. The risk of poor glycemic control increased by 4.3 times for patients diagnosed with type 2 diabetes and comorbid diseases (aOR: 4.33, 95% CI: 1.08–17.34). By contrast, a one year increasing in the age of the patients and those who were male were, respectively, 0.9 times (aOR: 0.95, 95% CI: 0.92–0.99) and 0.4 times (aOR: 0.39, 95% CI: 0.20–0.74) less likely to have poor glycemic control.

**Table 4:** Factors associated with poor glycemic control among outpatients with type 2 diabetes at an Indonesian clinic.

Variable	Glycemic control, *n* (%)		OR (95% CI)	adjusted OR (95% CI)
	Good	Poor		
Age (years)	198 (65.1)	106 (34.9)	0.95 (0.93–0.98)	0.95 (0.92–0.99)

Sex				
Female	99 (32.6)	68 (22.4)	1.00	
Male	99 (32.6)	38 (12.5)	0.56 (0.34–0.91)	0.39 (0.20–0.74)

Body mass index (kg/m^2^)				
<25.0	83 (34.3)	33 (13.6)	1.00	
≥25.0	78 (32.2)	48 (19.8)	1.55 (0.90–2.66)	1.84 (0.98–3.47)

Dietary adjustments				
Yes	162 (53.3)	93 (30.6)	1.00	
No	36 (11.8)	13 (4.3)	0.63 (0.32–1.25)	0.50 (0.20–1.29)

Duration of type 2 diabetes (years)	198 (65.1)	106 (34.9)	1.00 (0.97–1.03)	1.00 (0.96–1.05)

Number of prescribed medications				
<5	118 (38.8)	74 (24.3)	1.00	
≥5 (polypharmacy)	80 (26.3)	32 (10.5)	0.64 (0.39–1.05)	0.55 (0.27–1.12)

Diabetes medication				
Monotherapy	67 (22.0)	32 (10.5)	1.00	
Combination of oral hypoglycemic agents	69 (22.7)	28 (9.2)	0.85 (0.46–1.56)	2.00 (0.67–5.96)
Combination of oral hypoglycemic agents and insulin	62 (20.4)	46 (15.1)	1.55 (0.88–2.74)	2.74 (1.09–6.86)

Use of biguanide				
Yes	117 (38.5)	50 (16.4)	1.00	
No	81 (26.6)	56 (18.4)	1.62 (1.01–2.60)	2.73 (1.16–6.43)

Use of an antihyperlipidemic drug				
Yes	106 (34.9)	50 (16.4)	1.00	
No	92 (30.3)	56 (18.4)	1.29 (0.80–2.07)	1.57 (0.82–3.03)

Acute complication				
Hypoglycemia	32 (10.5)	20 (6.6)	1.55 (0.83–2.92)	0.90 (0.35–2.32)
Hyperglycemia	11 (3.6)	22 (7.2)	4.97 (2.27–10.85)	4.24 (1.53–11.81)
Both hypo- and hyperglycemia	1 (0.3)	2 (0.7)	4.97 (0.44–55.78)	7.96 (0.54–117.44)
No	154 (50.7)	62 (20.4)	1.00	

Presence of one or more comorbidities				
Yes	178 (58.6)	100 (32.9)	1.87 (0.73–4.82)	4.33 (1.08–17.34)
No	20 (6.6)	6 (2.0)	1.00	

Notes: Glycemic measurements from 304 patients were evaluated. The results of the Hosmer and Lemeshow”goodness-of-fit” test of the final model showed χ^2^ = 11.15 with degrees of freedom = 8. The model's discrimination, measured as the area under the receiver operating characteristic curve, was 0.79 (95% CI: 0.73–0.84).

CI = confidence interval; OR = odds ratio.

## Discussion

The findings of this study revealed that while the majority of outpatients with type 2 diabetes in the clinic under study did not adhere to their medication, two-thirds of these patients achieved their glycemic targets. The prevalence of medication nonadherence reported in this study was much higher than those reported in other studies using self-reporting questionnaires [18,19]. As reported elsewhere [20], forgetfulness was the most common self-reported reason for nonadherence.

In the present study, medication nonadherence was mostly reported for the regimen and recall screens. The majority of patients were prescribed combination therapy for their diabetes treatment, thus contributing to patient burden and difficulty remembering all the doses. Therefore, strategies that target poor adherence should focus on reducing treatment complexity and addressing patients' difficulties related to taking their medication. Interventions to eliminate self-reported nonadherence or barriers to drug use may include patient education or counseling by pharmacists to instruct or inform patients about treatment goals or to impart important information regarding their medications, including side effects, and devise schedules for medication intake [21,22]. Support provided by family and community members and peers has been reported to influence medication adherence and glycemic control [23].

Regular physical activity was found to be a predictor of medication nonadherence in this study. The finding that patients who did not engage in regular exercise was more likely to be adherent to medication was surprising, although the self-reported variable used in our finding may have had a limiting effect. Patients who exercise routinely could be controlling their weight or feeling healthier and may consequently demonstrate reduced adherence to their medication because of the fear of side effects or the feeling that taking medication is not necessary. For example, they could fear weight gain attributed to the use of insulin or sulphonylurea and reduce the doses or stop taking the medications, thereby leading to becoming less adherent. Conversely, a study in Malaysia found that less or no exercise was a strong determinant of nonadherence, but there was a lack supporting evidence of other healthy behaviors, such as dietary habits [24]. Given that a healthy lifestyle, including regular physical activity, is the cornerstone of type 2 diabetes management [1], further studies may be conducted to evaluate the relationship between regular physical activity and medication adherence and to explore the extent of the causal effect of physical activity. However, removing physical activity seems like an unlikely intervention in this situation unless extreme occupational demands are at issue.

The proportion of patients with poor glycemic control in the present study was similar to those reported in previous studies conducted in Iran [25] and Kenya [26] but was lower than those reported in studies conducted in Malaysia [7] and India [8]. This study found that poor glycemic control was associated with being female, aged younger, receiving a combination of oral hypoglycemic agents and insulin, not taking biguanide, and the presence of hyperglycemia and comorbid diseases. Previous studies conducted in Malaysia, Brazil, and Venezuela also reported similar results, notably that glycemic control was poorer among women than among men [8,27]. This study's findings on age status is consistent with those of other studies performed in Denmark and Malaysia [[6], [7], [8]]. Conversely, a study conducted in Ethiopia found that poor glycemic control was more prevalent among older patients [28]. Patients who had one or more comorbid conditions had higher A1C levels; a finding that is consistent with those of previous studies [29,30]. Disturbances relating to insulin metabolism may be compounded in patients who develop complications or have comorbidities, leading to poor blood glucose regulation.

The finding of an association between patients who were receiving a combination of oral hypoglycemic agents and insulin and poorly A1C controlled implied that multitherapy might not provide satisfactory glycemic control. Although this combination is recommended if patients do not achieve an A1C level lower than 7%, the poor glycemic outcome among patients receiving it could probably be as a result of the increasing difficulty in taking more than one drug and the injection [7]. Thus, patients treated with a combination of insulin and oral hypoglycemic agents required more aggressive treatment and monitoring in terms of adequate dosing and improved adherence to achieve better glycemic outcome. Furthermore, patients who were not taking biguanide were more likely to have inadequate glycemic control than those receiving it. Biguanide (e.g., metformin) is widely used as first-line therapy of type 2 diabetes and effective as monotherapy and in combination with other diabetes medications. Metformin therapy has been shown to improve glycemic control and reduce hypoglycemic events and weight gain compared with sulfonylurea and insulin [31]. However, its gastrointestinal side effects, such as nausea, flatulence, and diarrhea, may prevent patients from taking it, leading to inadequate blood glucose control. Reasons for failure to achieve the desired glycemic outcome in this group of patients need to be further explored.

Medication adherence is known to be a factor affecting A1C levels. Many studies have demonstrated that high adherence is associated with lower levels of A1C irrespective of the method to measure adherence [5,7,19,20]. Nevertheless, this study did not find an association between adherence and glycemic control. A similar result was also reported in another study conducted in Iran [18]. This discrepancy could be attributed to different methods used to measure adherence. The present study used a self-reported questionnaire to assess adherence, which could be prone to recall bias. Further studies using more objective methods, including measuring the quantities of drugs present in body fluids using plasma or alternative, less invasive matrices [32], could be used to obtain more granular adherence data. In the present study, although self-care behaviors did not appear to influence glycemic control, patients should be consistently advised to control their intake of particular foods, exercise regularly, and stop smoking. Furthermore, as the duration of diabetes increases, patients become negligent in adhering to their medication and sustaining their glycemic control measures [33]. Chronic complications and numerous episodes of acute complications are associated with a longer duration of diabetes. For better outcomes, other chronic illnesses should also be addressed in interventions.

We evaluated many variables related to patients' characteristics, disease management, and medication used to treat diabetes. Nonetheless, the study has some limitations, and the results should be interpreted with caution. The generalizability of the findings may be limited because this study was performed at a single center and prone to selection bias. Patients were recruited directly at the hospital-based clinic; only patients who actively went to the clinic participated to the study, thus other eligible patients might have been missed because of being too sick or busy to attend appointments. A direct causal relationship between predictors and treatment outcomes could not be explained using a cross-sectional design. The study only captured the exposures and outcomes from the patients with type 2 diabetes who were alive and visited the clinic and left out those who were too sick or had passed away. Some exposures e.g. acute or chronic complications and comorbidities, might decrease the survival of those patients, thus the outcomes could not be observed. This selective survival bias might influence the estimate odds ratios of these predictors [34,35]. A longitudinal cohort or case-control study may be beneficial in this setting as the dynamic process of adherence and glycemic control that may change over time and their causal factors could be further assessed [35].

Another important limitation of the study is related to the backward elimination used to result a final model with variables important for prediction. Although this approach is preferred if stepwise selection is performed, it is prone to testimation bias and events per variable (EPV) > 50 may be required for selecting predictors from a larger set of candidate predictors [36]. Furthermore, the study still dichotomized some continuous variables, e.g. body mass index and number of prescribed medications, as it was inevitable due to the nature of collected data. The categorization might decrease the statistical power to detect a relation between the variable and the outcome, underestimate the extent of variation in outcome between the groups, and result in the residual confounding effect [37]. Thus, it is recommended to keep the quantitative variables continuous in the future similar studies; however, if grouping is needed, fractional polynomials and spline approaches could be performed to obtain the best scale [38]. Moreover, patients' health literacy and any knowledge gaps relating to diabetes and its management, which could contribute to lower medication adherence, were not assessed. At the time of conducting this study, Indonesia's national health insurance scheme did not cover new types of hypoglycemic agents, such as meglitinides, glucagon-like peptide-1 agonists, dipeptidyl peptidase 4 inhibitors, and sodium-glucose cotransporter-2 inhibitors. Therefore, glycemic outcomes for patients using these classes of medication should be investigated in future studies.

## Conclusions

Nonadherence to diabetes medication was found to be more prevalent than poor glycemic control among outpatients with type 2 diabetes at the Indonesian clinic where this study was conducted. Regular physical activity was associated with medication nonadherence. The age and sex of a patient, diabetes medication, not taking biguanide, and the occurrence of acute complications and comorbidities were associated with poor glycemic control. Therefore, strategies considering these influencing factors are warranted to improve medication adherence and achieve the desired glycemic outcomes.

## Funding

This study was funded by Universitas Airlangga, Surabaya, Indonesia, through a research grant of *Penelitian Unggulan Fakultas* 2018. However, the funding body did not involve in the design of the study, data collection and analysis, and in writing the manuscript.

## CRediT authorship contribution statement

**Budi Suprapti:** Conceptualization, Funding acquisition, Investigation, Methodology, Supervision, Validation, Writing – review & editing. **Zamrotul Izzah:** Conceptualization, Data curation, Formal analysis, Investigation, Project administration, Visualization, Writing – original draft. **Ade Giriayu Anjani:** Formal analysis, Project administration, Visualization, Writing – original draft. **Mareta Rindang Andarsari:** Conceptualization, Investigation, Project administration, Visualization, Writing – review & editing. **Wenny Putri Nilamsari:** Formal analysis, Project administration, Validation, Visualization, Writing – review & editing. **Cahyo Wibisono Nugroho:** Conceptualization, Investigation, Methodology, Supervision, Writing – review & editing.

## Declaration of competing interest

The authors have no financial or non-financial interests to declare that are relevant to the content of this article.

## Appendix A Supplementary data

Supplementary text and tables Image 1
