# Supplementary material for: Prevalence of medication adherence and glycemic control among patients with type 2 diabetes and influencing factors: A cross-sectional study
Source: Glob Epidemiol. 2023 Jun 10;5:100113. doi: 10.1016/j.gloepi.2023.100113 (PMC10446000; doi:10.1016/j.gloepi.2023.100113)
Supplement: Supplementary file 1 — Supplementary text and tables [file mmc1.docx]

**Supplementary Text**

**Bahasa Indonesia version of the Brief Medication Questionnaire (BMQ)**

**Kuesioner Penggunaan Obat**

Kode Responden:

1. Silahkan tuliskan **semua obat** yang Anda gunakan dalam **seminggu terakhir**. Untuk setiap obat yang dituliskan, tolong jawab pertanyaan berikut:

| a. Nama obat dan kekuatan | b. Berapa hari obat telah digunakan? | c. Berapa kali obat digunakan dalam sehari? | d. Berapa jumlah obat untuk setiap penggunaan? | e. Berapa kali terlupa menggunakan obat? | f. Apa alasan penggunaan obat? | g. Seberapa baik obat tersebut bekerja pada Anda?  1 = baik  2 = cukup  3 = tidak baik |
| --- | --- | --- | --- | --- | --- | --- |
|  |  |  |  |  |  |  |
|  |  |  |  |  |  |  |
|  |  |  |  |  |  |  |
|  |  |  |  |  |  |  |
|  |  |  |  |  |  |  |

2. Apakah obat yang Anda gunakan tersebut mengganggu Anda? Ya Tidak

a. Bila **Ya**, tolong tuliskan nama obat dan pilih seberapa mengganggu obat tersebut:

| Nama Obat | Seberapa mengganggu obat tersebut pada Anda? | | | | |
| --- | --- | --- | --- | --- | --- |
|  | Sangat | Cukup | Sedikit | Tidak pernah | Bagaimana obat tersebut mengganggu anda? |
|  |  |  |  |  |  |
|  |  |  |  |  |  |
|  |  |  |  |  |  |
|  |  |  |  |  |  |
|  |  |  |  |  |  |

3. Berikut adalah daftar masalah yang sering dihadapi orang-orang terhadap obat yang digunakan. Tolong beri tanda **√** seberapa sulit Anda melakukan hal tersebut:

|  | Sangat sulit | Terkadang sulit | Tidak sulit sama sekali | Nama Obat |
| --- | --- | --- | --- | --- |
| a. Membuka atau menutup wadah obat |  |  |  |  |
| b. Membaca label atau etiket pada wadah |  |  |  |  |
| c. Mengingat untuk menggunakan semua obat |  |  |  |  |
| d. Menebus resep ulang tepat waktu |  |  |  |  |
| e. Menggunakan banyak obat dalam satu waktu |  |  |  |  |

4. Apakah pernah berhenti minum obat karena terlambat kontrol atau alasan lain? Ya Tidak

5. Apakah pernah mengurangi sendiri takaran/dosis obat yang digunakan tanpa sepengetahuan dokter? Ya Tidak

6. Apakah pernah menggunakan obat lebih dari frekuensi penggunaan yang disarankan? Ya Tidak

*Adapted with permission from the original copyrighted instrument (in English) developed by Svarstad BL, Chewning BA, Sleath BL, et al. The Brief Medication Questionnaire : A tool for screening patient adherence and barriers to adherence. Patient Educ Couns. 1999;37:113–24.*

**Supplementary Table S1. Bivariate analysis of variable associated with medication nonadherence among outpatients with type 2 diabetes**

| **Variable** | **Adherence level, n (%)** | | **Odds ratio**  **(95% CI)** |
| --- | --- | --- | --- |
|  | **Adherent** | **Nonadherent** |  |
| **Age (years)** | 53 (16.5) | 268 (83.5) | 0.99  (0.96–1.02) |
| **Sex** | | | |
| Female | 22 (6.9) | 149 (46.4) | 1.00 |
| Male | 31 (9.7) | 119 (37.1) | 0.57  (0.31–1.03) |
| **Body mass index (kg/m^2^)** | | | |
| <25.0 | 20 (7.8) | 102 (39.8) | 1.00 |
| ≥25.0 | 20 (7.8) | 114 (44.5) | 1.12  (0.57–2.20) |
| **Education level** | | | |
| Tertiary | 15 (4.8) | 53 (17.0) | 1.00 |
| Secondary | 28 (9.0) | 152 (48.9) | 1.54  (0.76–3.10) |
| Primary | 8 (2.6) | 55 (17.7) | 1.95  (0.76–4.97) |
| **Currently smoking** | | | |
| Yes | 4 (1.2) | 21 (6.5) | 1.04  (0.34–3.17) |
| No | 49 (15.3) | 247 (76.9) | 1.00 |
| **Dietary adjustments** | | | |
| Yes | 41 (12.8) | 229 (71.3) | 1.00 |
| No | 12 (3.7) | 39 (12.1) | 0.58  (0.28–1.20) |
| **Regular physical activity** | | | |
| Yes | 25 (7.8) | 167 (52.0) | 1.00 |
| No | 28 (8.7) | 101 (31.5) | 0.54  (0.30–0.98) |
| **Duration of type 2 diabetes (years)** | 53 (16.5) | 268 (83.5) | 0.99  (0.96–1.03) |
| **Number of prescribed medications** | | | |
| <5 | 31 (9.7) | 172 (53.6) | 1.00 |
| ≥5 | 22 (6.9) | 96 (29.9) | 0.79  (0.43–1.43) |
| **Diabetes medication** | | | |
| Monotherapy | 16 (5.0) | 88 (27.4) | 1.00 |
| Combination of oral hypoglycemic agents | 12 (3.7) | 91 (28.3) | 1.38  (0.62–3.08) |
| Combination of oral hypoglycemic agents and insulin | 25 (7.8) | 89 (27.7) | 0.65  (0.32–1.30) |
| **Use of insulin** | | | |
| Yes | 36 (11.2) | 155 (48.3) | 0.65  (0.35–1.21) |
| No | 17 (5.3) | 113 (35.2) | 1.00 |
| **Use of biguanide** | | | |
| Yes | 30 (9.3) | 149 (46.4) | 0.96  (0.53–1.74) |
| No | 23 (7.2) | 119 (37.1) | 1.00 |
| **Use of sulphonylurea** | | | |
| Yes | 27 (8.4) | 143 (44.5) | 1.10  (0.61–1.99) |
| No | 26 (8.1) | 125 (38.9) | 1.00 |
| **Use of an alpha glucosidase inhibitor** | | | |
| Yes | 10 (3.1) | 72 (22.4) | 1.58  (0.75–3.31) |
| No | 43 (13.4) | 196 (61.1) | 1.00 |
| **Use of thiazolidinedione** | | | |
| Yes | 3 (0.9) | 4 (1.2) | 0.25  (0.06–1.16) |
| No | 50 (15.6) | 264 (82.2) | 1.00 |
| **Use of an antihypertensive agent** | | | |
| Yes | 31 (9.7) | 154 (48.0) | 0.96  (0.53–1.74) |
| No | 22 (6.9) | 114 (35.5) | 1.00 |
| **Use of an antihyperlipidemic drug** | | | |
| Yes | 31 (9.7) | 133 (41.4) | 0.70  (0.39–1.27) |
| No | 22 (6.9) | 135 (42.1) | 1.00 |
| **Use of a platelet-aggregation inhibitor** | | | |
| Yes | 17 (5.3) | 67 (20.9) | 0.71  (0.37–1.34) |
| No | 36 (11.2) | 201 (62.6) | 1.00 |
| **Acute complication** | | | |
| Hypoglycemia | 12 (3.7) | 43 (13.4) | 0.68  (0.33–1.41) |
| Hyperglycemia | 4 (1.2) | 32 (10.0) | 1.51  (0.50–4.52) |
| Both hypo- and hyperglycemia | 1 (0.3) | 2 (0.6) | 0.38  (0.03–4.27) |
| No | 36 (11.2) | 191 (59.5) | 1.00 |
| **Presence of one or more comorbidities** | | | |
| Yes | 51 (15.9) | 243 (75.7) | 0.38  (0.09–1.66) |
| No | 2 (0.6) | 25 (7.8) | 1.00 |
| **Presence of a kidney disease** | | | |
| Yes | 9 (2.8) | 32 (10.0) | 0.66  (0.30–1.49) |
| No | 44 (13.7) | 236 (73.5) | 1.00 |

**Note**: Data were analyzed from 321 patients.

CI=confidence interval

**Supplementary Table S2. Bivariate analysis of variable associated with poor glycemic control among outpatients with type 2 diabetes**

| **Variable** | **Glycemic control, n (%)** | | **Odds ratio**  **(95% CI)** | |
| --- | --- | --- | --- | --- |
|  | **Good** | **Poor** |  |  |
| **Age (years)** | 198 (65.1) | 106 (34.9) | 0.95  (0.93–0.98) | |
| **Sex** | | | |  |
| Female | 99 (32.6) | 68 (22.4) | 1.00 | |
| Male | 99 (32.6) | 38 (12.5) | 0.56  (0.34–0.91) | |
| **Body mass index (kg/m^2^)** | | | |  |
| <25.0 | 83 (34.3) | 33 (13.6) | 1.00 | |
| ≥25.0 | 78 (32.2) | 48 (19.8) | 1.55  (0.90–2.66) | |
| **Education level** | | | |  |
| Tertiary | 38 (12.9) | 24 (8.2) | 1.00 | |
| Secondary | 121 (41.2) | 51 (17.3) | 0.67  (0.36–1.22) | |
| Primary | 35 (11.9) | 25 (8.5) | 1.13  (0.55–2.33) | |
| **Currently smoking** | | | |  |
| Yes | 20 (6.6) | 5 (1.6) | 0.44  (0.16–1.21) | |
| No | 178 (58.6) | 101 (33.2) | 1.00 | |
| **Dietary adjustments** | | | |  |
| Yes | 162 (53.3) | 93 (30.6) | 1.00 | |
| No | 36 (11.8) | 13 (4.3) | 0.63  (0.32–1.25) | |
| **Regular physical activity** | | | |  |
| Yes | 113 (37.2) | 69 (22.7) | 1.00 | |
| No | 85 (28.0) | 37 (12.2) | 0.71  (0.44–1.16) | |
| **Duration of type 2 diabetes (years)** | 198 (65.1) | 106 (34.9) | 1.00  (0.97–1.03) | |
| **Number of prescribed medications** | | | |  |
| <5 | 118 (38.8) | 74 (24.3) | 1.00 | |
| ≥5 | 80 (26.3) | 32 (10.5) | 0.64  (0.39–1.05) | |
| **Diabetes medication** | | | |  |
| Monotherapy | 67 (22.0) | 32 (10.5) | 1.00 | |
| Combination of oral hypoglycemic agents | 69 (22.7) | 28 (9.2) | 0.85  (0.46–1.56) | |
| Combination of oral hypoglycemic agents and insulin | 62 (20.4) | 46 (15.1) | 1.55  (0.88–2.74) | |
| **Use of insulin** | | | |  |
| Yes | 108 (35.5) | 74 (24.3) | 1.00 | |
| No | 90 (29.6) | 32 (10.5) | 0.52  (0.32–0.86) | |
| **Use of biguanide** | | | |  |
| Yes | 117 (38.5) | 50 (16.4) | 1.00 | |
| No | 81 (26.6) | 56 (18.4) | 1.62  (1.01–2.60) | |
| **Use of sulphonylurea** | | | |  |
| Yes | 107 (35.2) | 53 (17.4) | 1.00 | |
| No | 91 (29.9) | 53 (17.4) | 1.18  (0.73–1.89) | |
| **Use of an alpha glucosidase inhibitor** | | | |  |
| Yes | 45 (14.8) | 33 (10.9) | 1.00 | |
| No | 153 (50.3) | 73 (24.0) | 0.65  (0.38–1.10) | |
| **Use of thiazolidinedione** | | | |  |
| Yes | 4 (1.3) | 3 (1.0) | 1.00 | |
| No | 194 (63.8) | 103 (33.9) | 0.71  (0.16–3.22) | |
| **Use of an antihypertensive agent** | | | |  |
| Yes | 112 (36.8) | 62 (20.4) | 1.00 | |
| No | 86 (28.3) | 44 (14.5) | 0.92  (0.57–1.49) | |
| **Use of an antihyperlipidemic drug** | | | |  |
| Yes | 106 (34.9) | 50 (16.4) | 1.00 | |
| No | 92 (30.3) | 56 (18.4) | 1.29  (0.80–2.07) | |
| **Use of a platelet-aggregation inhibitor** | | | |  |
| Yes | 55 (18.1) | 23 (7.6) | 1.00 | |
| No | 143 (47.0) | 83 (27.3) | 1.39  (0.80–2.42) | |
| **Acute complication** | | | |  |
| Hypoglycemia | 32 (10.5) | 20 (6.6) | 1.55  (0.83–2.92) | |
| Hyperglycemia | 11 (3.6) | 22 (7.2) | 4.97  (2.27–10.85) | |
| Both hypo- and hyperglycemia | 1 (0.3) | 2 (0.7) | 4.97  (0.44–55.78) | |
| No | 154 (50.7) | 62 (20.4) | 1.00 | |
| **Presence of one or more comorbidities** | | | |  |
| Yes | 178 (58.6) | 100 (32.9) | 1.87  (0.73–4.82) | |
| No | 20 (6.6) | 6 (2.0) | 1.00 | |
| **Presence of kidney disease** | | | |  |
| Yes | 29 (9.5) | 11 (3.6) | 0.68  (0.32–1.41) | |
| No | 169 (55.6) | 95 (31.3) | 1.00 | |
| **Adherence level** | | | |  |
| Adherent | 31 (10.2) | 17 (5.6) | 1.00 | |
| Nonadherent | 167 (54.9) | 89 (29.3) | 0.97  (0.51–1.85) | |

**Note**: Glycemic measurements from 304 patients' data were evaluated.

CI=confidence interval
